# Supplementary material for: Clinical Features and Outcomes of Enterococcal Bone and Joint Infections and Factors Associated with Treatment Failure over a 13-Year Period in a French Teaching Hospital
Source: Microorganisms. 2023 May 5;11(5):1213. doi: 10.3390/microorganisms11051213 (PMC10220579; doi:10.3390/microorganisms11051213)
Supplement: Supplementary file 1 [file microorganisms-11-01213-s001.zip › microorganisms-2374002-supplementary.pdf]

Supplementary materials

Clinical features and outcomes of enterococcal bone and joint infections and factors associated with treatment failure over a 13-year period in a French teaching hospital

Aurélie Martin, Paul Loubet, Florian Salipante, Paul Laffont-Lozes, Julien Mazet, Jean-Philippe Lavigne, Nicolas Cellier, Albert Sotto, Romaric Larcher

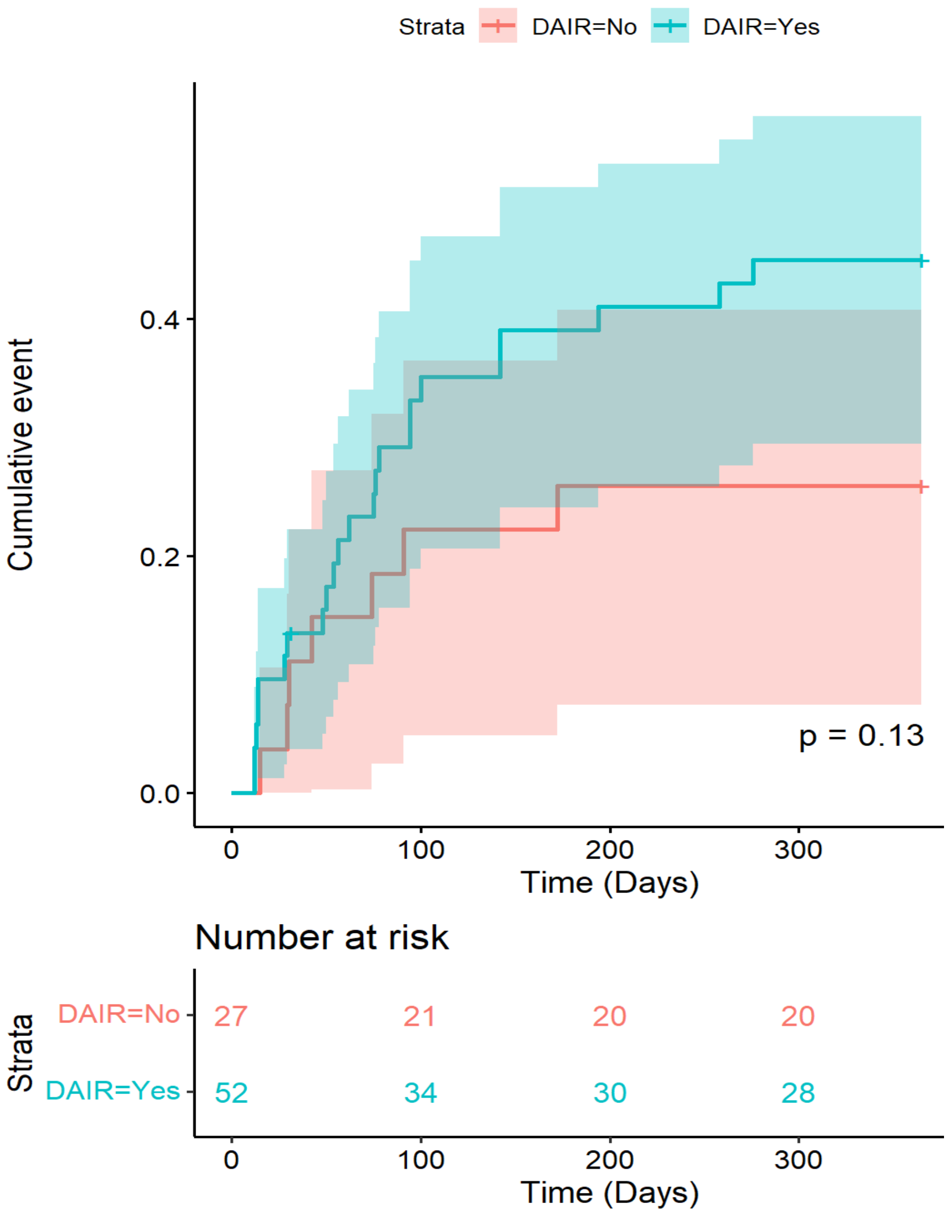

Figure S1. Cumulative incidence curves of treatment failure in patients with surgical debridement, antibiotic, and implant retention, also known as DAIR (green) and in those with implant removal (red) for enterococcal prosthetic joint infections or orthopedic implant-associated infections.

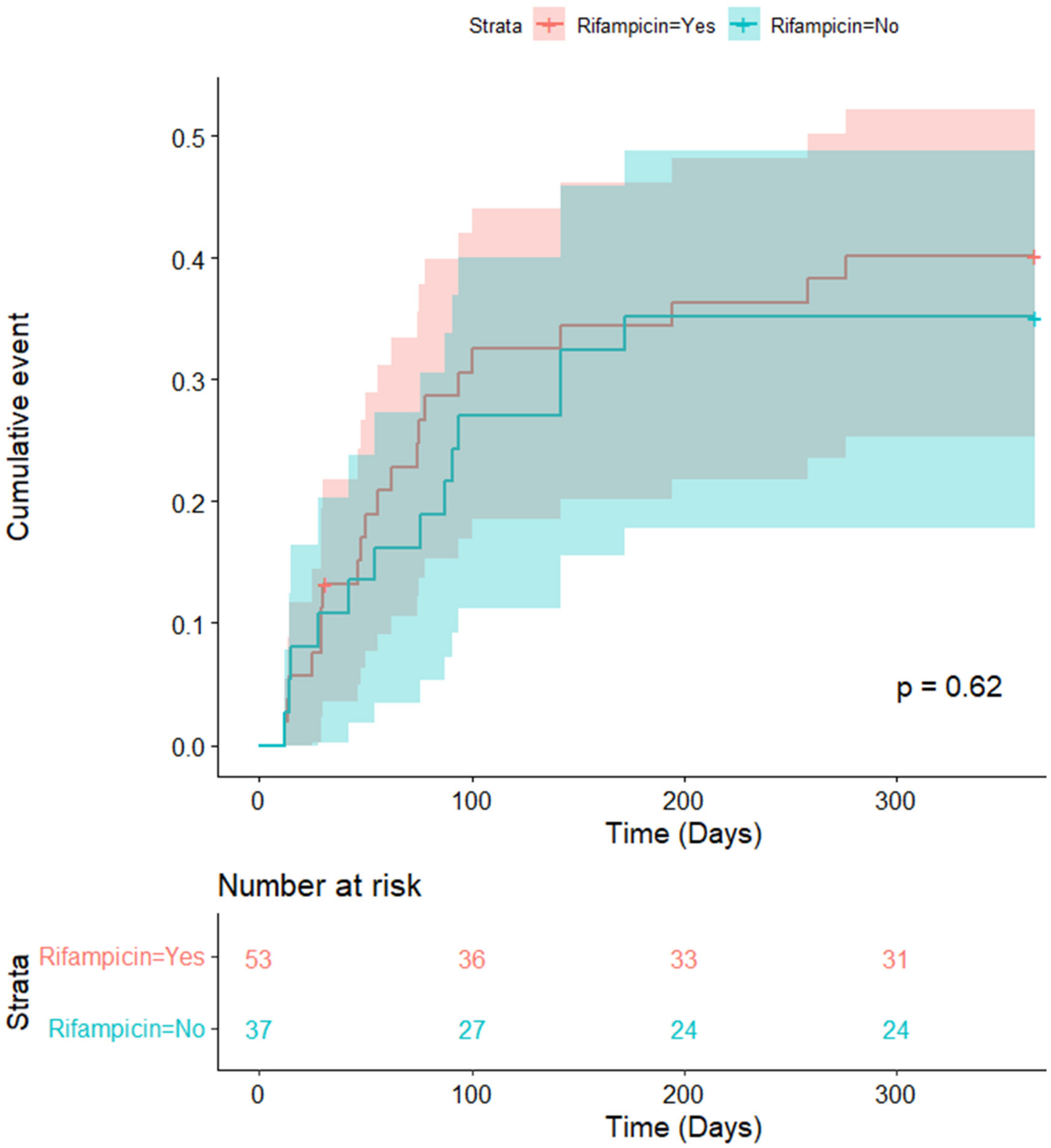

Figure S2. Cumulative incidence curves of treatment failure in patients with (red) or without (green) rifampicin combination therapy for enterococcal bone and joint infections.
